# Supplementary material for: Longitudinal genomic surveillance of multidrug-resistant Escherichia coli carriage in critical care patients
Source: Microbiol Spectr. 2024 Jan 3;12(2):e03128-23. doi: 10.1128/spectrum.03128-23 (PMC10846182; doi:10.1128/spectrum.03128-23)
Supplement: Supplemental material — Figures S1 to S4 and Tables S1 to S3. [file spectrum.03128-23-s0001.docx]

Longitudinal genomic surveillance of multidrug-resistant *Escherichia coli* carriage

in critical care patients.

**Supplemental Material**

This paper is supported by a total of four Supplementary Figures and three Supplementary Tables which are listed below following the numerical sequence of those in the main text.

**Supplementary Figure S1**: **Boxplots displaying the major allele frequency distributions** (i.e., frequency of higher allele, whether alternative or reference) based on single-nucleotide variants and insertions/deletions. These variants were called by Snippy, based on mapped reads to a reference *Escherichia* *coli* genome. Samples are separated by whether they passed an independent quality control step of whether the whole-genome assembly was >= 8 megabase pairs (Mbp), indicating a potential mixture of strains or other contamination. The number of variants per sample ranges from 8,971-82,636 (mean: 45,920). The box within each boxplots represents the median (centre line) and the 25th and 75th percentiles as the box edges. The whiskers represent 1.5 * inter-quartile range (i.e., the range between the 25th and 75th percentiles), or to the most extreme value. Points indicate outliers outside this range.


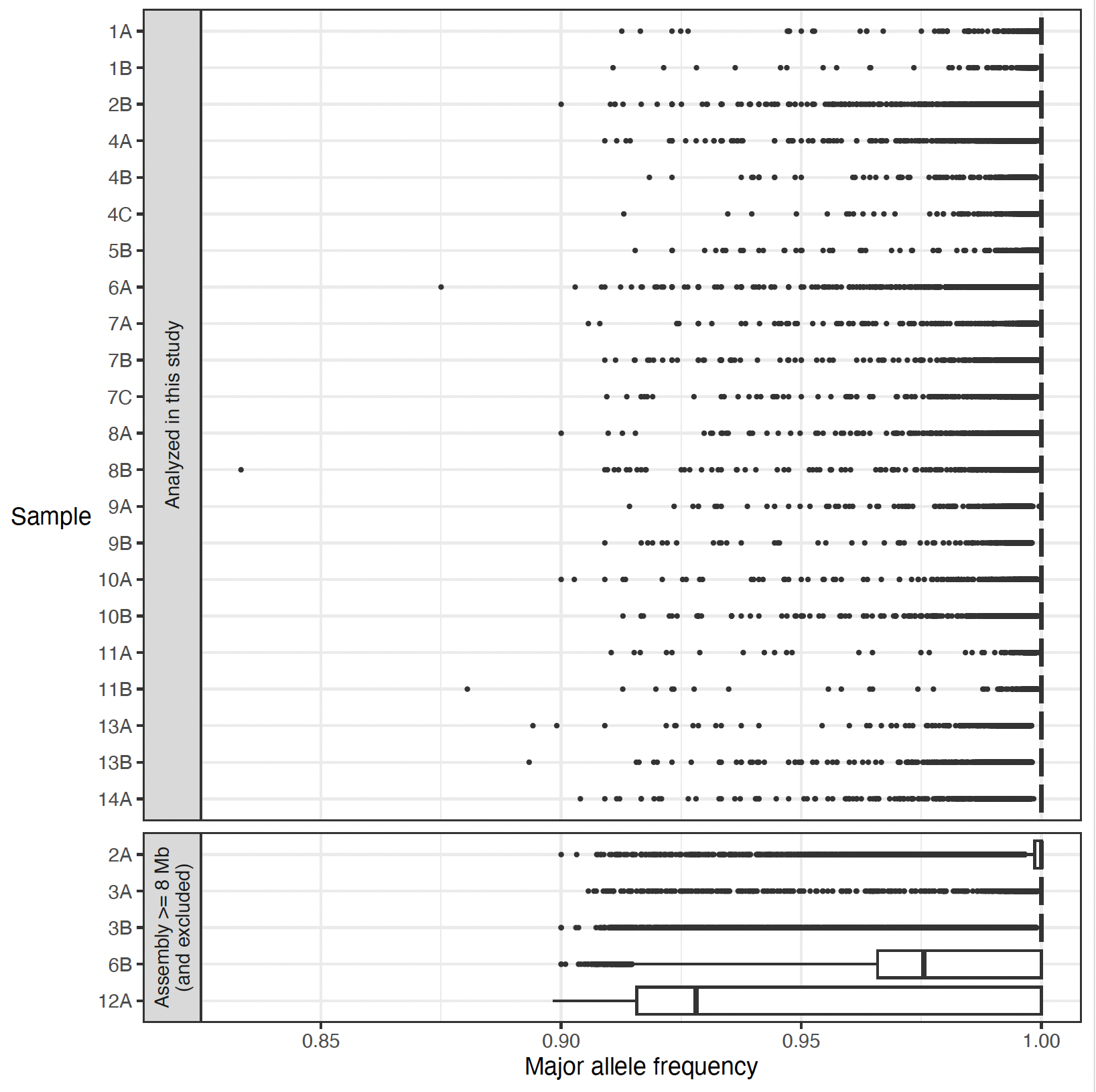


# Supplementary Figure S2: Heatmap summary of Average Nucleotide Identity between each of the 22 strains sequenced in this study. Values range from 96% ANI to 100% ANI.


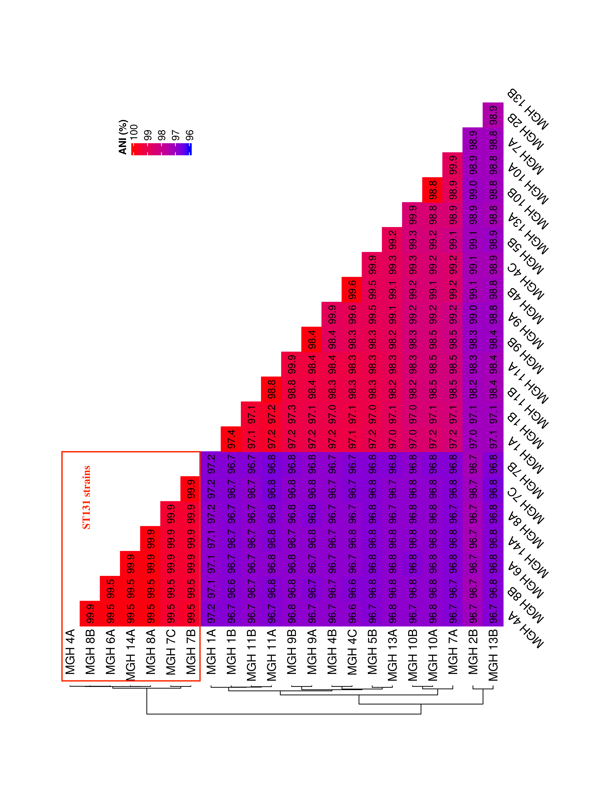


# Supplementary Figure S3: Heatmap showing the plasmid types identified in each of 22 *E. coli* genomes. The x axis represents the plasmid types.

# .


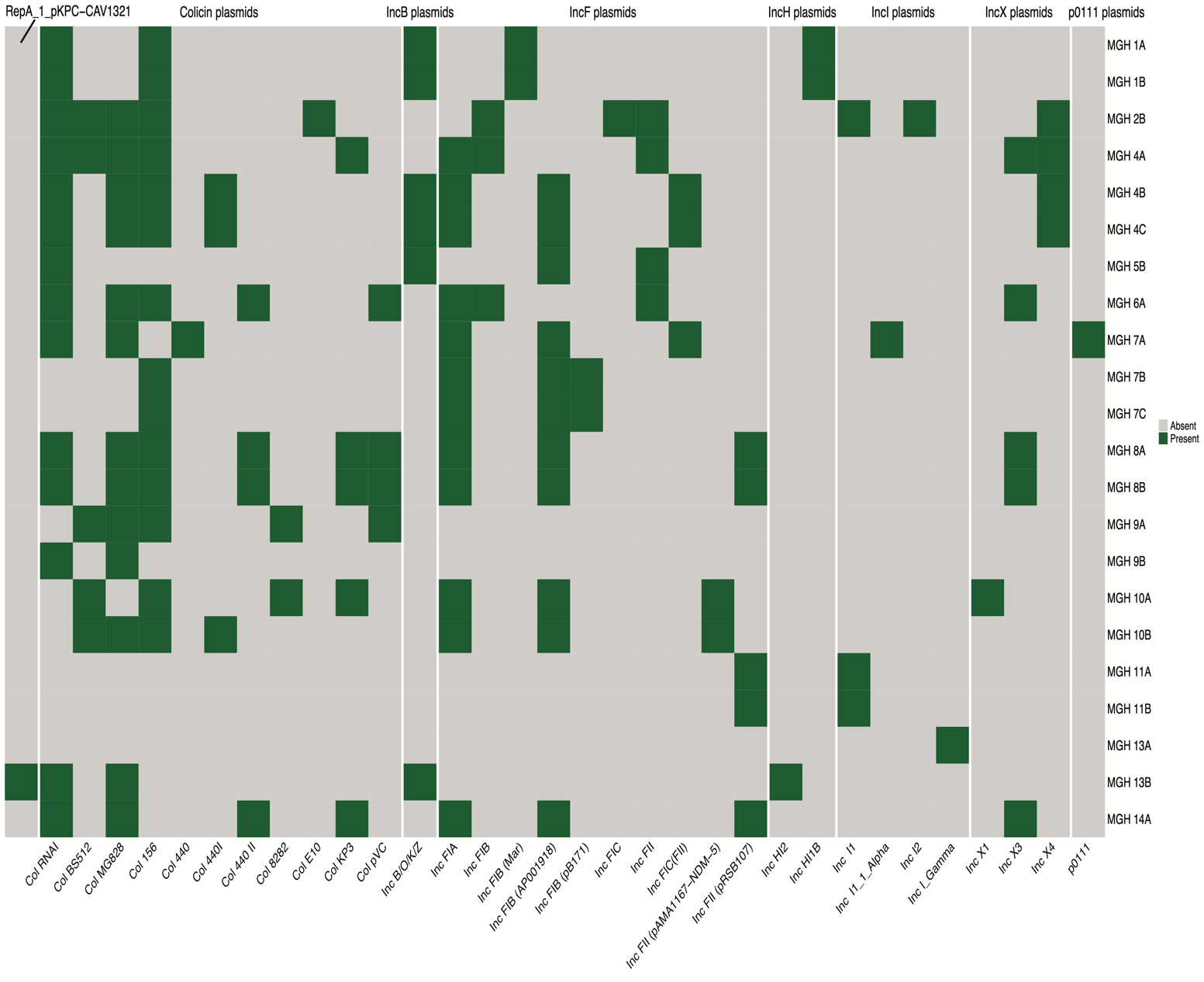


**Supplementary Figure S4: Heatmap showing the virulence factors identified in each of 22 *E. coli* genomes.** The x axis represents the virulent genes identified using ABRicate.


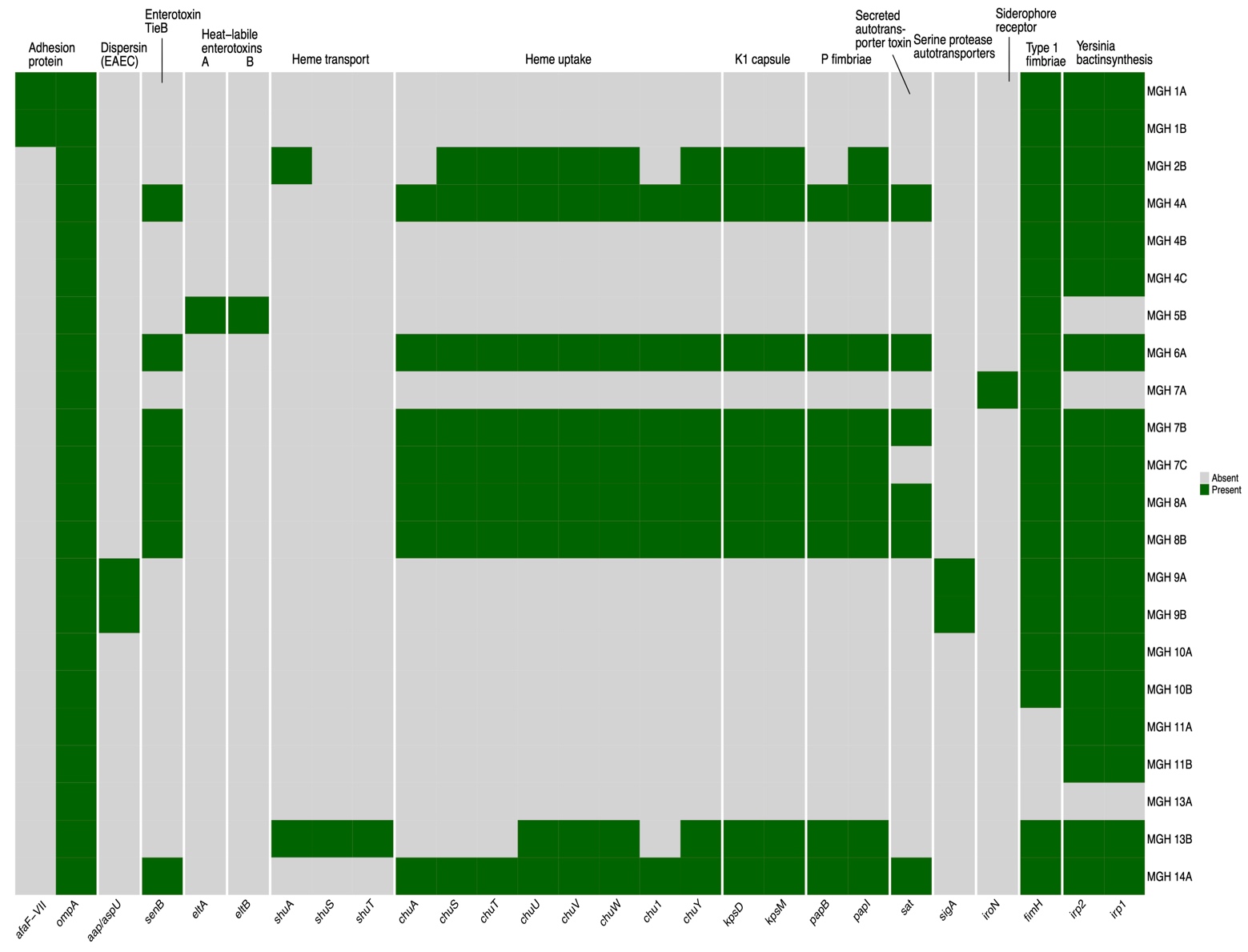


**Supplementary Table S1: Demographic and Clinical characteristics of 14 patients enrolled in the study.** The table includes demographic data in addition to the hospitalization status, risk factors, and antibiotic use during hospitalization. N/A refers to Not Available.


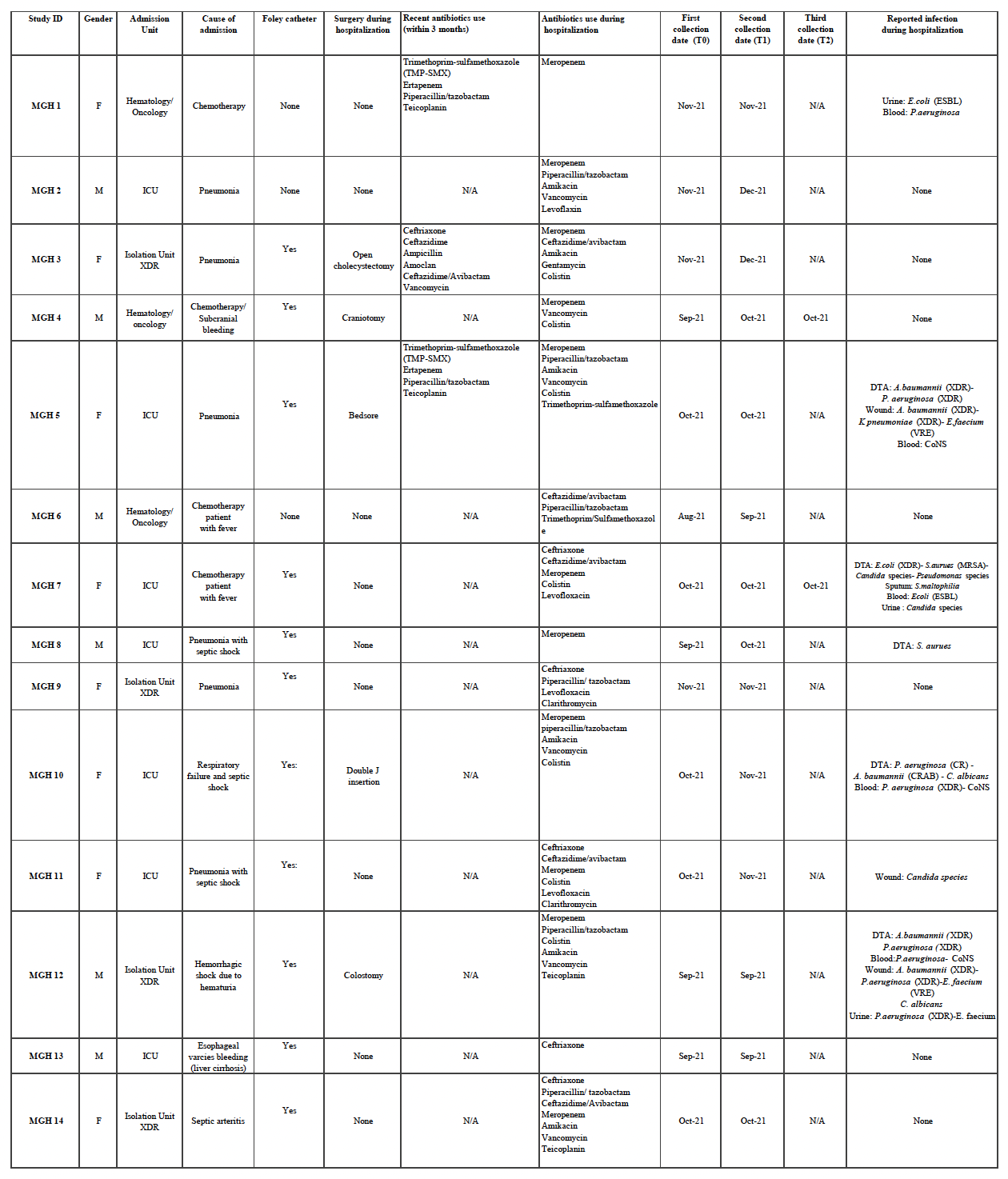


**Supplementary Table S2: Antimicrobial susceptibility testing results of 32 isolates identified in 15 patients using the disk diffusion method.**


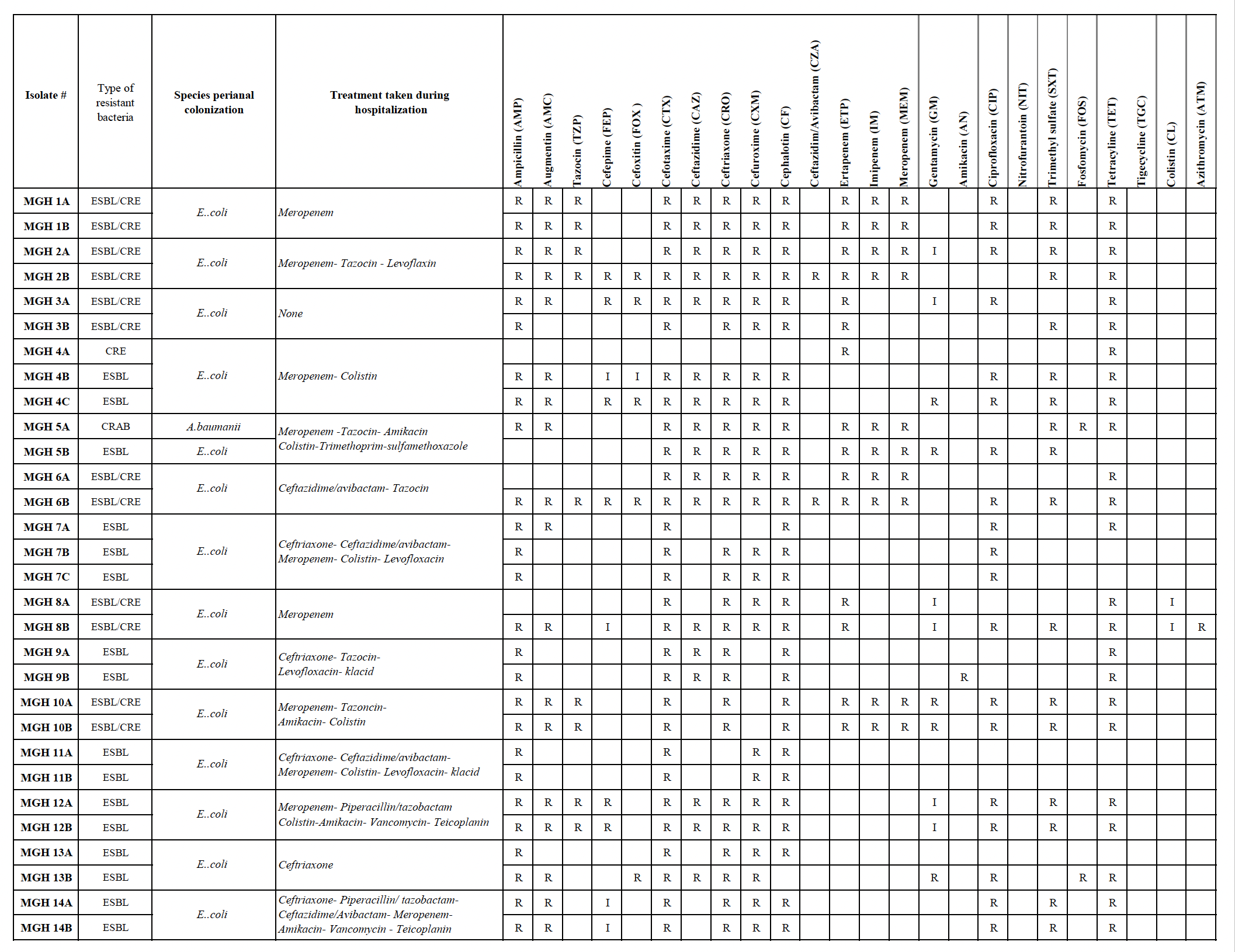


**Supplementary Table S3: General characteristics of genetic variants identified in seven patients at two different time points.**
